# Self-determination theory in ophthalmology education: factors influencing autonomy, competence and relatedness in medical students

**DOI:** 10.1080/10872981.2023.2258633

**Published:** 2023-09-20

**Authors:** Deepaysh D.C.S. Dutt, Hessom Razavi, Sandra E. Carr

**Affiliations:** a Health Professions Education, The University of Western Australia, Perth, Western Australia, Australia; b Centre for Ophthalmology and Visual Science, The University of Western Australia, Perth, Western Australia, Australia; c Lions Eye Institute, Department of Ophthalmology, Perth, Western Australia, Australia

**Keywords:** Medical education, motivation, ophthalmology, Self-determination theory, phenomenography

## Abstract

**Background:**

The affective components of learning, including student motivation, has yet to be thoroughly investigated in undergraduate ophthalmology education. This study aims to use Self-Determination Theory (SDT) as a framework to describe the variations in student perceptions of motivation in studying ophthalmology through their satisfactions of autonomy, competence and relatedness, and to highlight factors that stimulate or hinder this.

**Methods:**

Penultimate year medical students from a single tertiary educational institution undertaking a clinical placement in ophthalmology participated in in-depth interviews to explore factors affecting their perceptions of motivation in studying ophthalmology. Interviews were transcribed and analysed according to the principles of interpretive phenomenography through the theoretical framework of SDT.

**Results:**

Of the 39 students invited, 10 agreed to participate. Variations in perceptions of experiences generated the outcome space. Participants experienced either amotivation, external locus extrinsic motivation, internal locus extrinsic motivation and intrinsic motivation (conceptions of the outcome space). This was described with respect to their satisfaction of autonomy, competence and relatedness (dimensions of the outcome space). Additionally, 21 factors that impacted on motivation were identified, of which five over-arching factors impacted all three basic psychological needs – guidance, growth mindset, assessment, curricular pressure and extracurricular pressure.

**Conclusions:**

The findings of this study provide a unique insight into the motivation of medical students studying ophthalmology. This provides an exciting opportunity for medical educators to address the affective aspect of learning.

## Background

As many as 19% of patients presenting to a general practice do so due to underlying ophthalmic disease [1–3]. However, the presence of ophthalmology content and clinical experience has been declining in medical schools worldwide. Since 2014, fewer than 20% of medical schools require an ophthalmology clerkship in the US and Canada [4,5]. A similar experience has been occurring in the UK, where variations in ophthalmic teaching have resulted in many schools not meeting education recommendations made by the International Council of Ophthalmology (ICO) [6,7]. In addition, a study by Fan et al. [2] showed reductions or even the absence of ophthalmic teaching in Australian medical schools, with a great variation in curriculum outcomes reported. This downward trend in teaching time and content has been exacerbated by the impact of the COVID-19 pandemic [8,9]. The limited time and resources devoted to ophthalmic teaching in medical schools has inevitably culminated in lower levels of confidence and preparedness in medical graduates when diagnosing and managing ophthalmic disease and presentations [10–14].

Current efforts to address these concerns have focused on increasing the time allocated to undergraduate ophthalmic education and improving the efficiency of delivering content. Initiatives such as peer assisted learning activities [15] and team-based learning activities [16] have been implemented with some reported success. Trends in medical education favouring self-directed learning and problem-based learning have also been successfully implemented in ophthalmology teaching in medical schools [17–19]. In addition, the adaptation of recent technological advancements in medical education have proven beneficial. These include virtual reality simulations [19], virtual clinics with case based learning [20–22] and eLearning modules [23].

However, one aspect of medical education that is yet to be studied with regards to ophthalmic education is student motivation. Motivation is defined as the reason behind thought and behaviour [24]. Student motivation (why we learn), along with cognitive (what to learn) and metacognitive (how to learn) regulation, are described in educational psychology as the three dimensions of the learning process [25,26]. Multiple models have been developed to conceptualise motivation in students, however one model considered useful when studying medical student motivation is the Self-Determination Theory of Motivation (SDT) [26–29].

**Figure 1.**
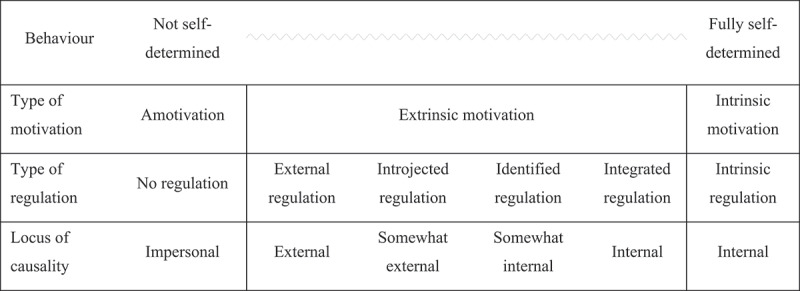
Relationship between types of motivation and locus of causality according to self-determination theory.

SDT was empirically developed by Edward Deci and Richard Ryan [30–32]. SDT proposes that student behaviour and educational outcomes are not a product of their amount of motivation, rather their quality of motivation. SDT follows the principle that human behaviour is determined through intrinsic or extrinsic motivation, as conceptualised in Figure 1 [33]. Intrinsic motivation describes individuals who are driven to act out of their inherent interest in a behaviour or task and not in response to external factors, such as seeking rewards or avoiding punishment [33]. Extrinsic motivation describes thought and action as a response to external stimuli, generally to attain reward or avoid punishment [33]. Extrinsic motivation is regulated by factors external to one’s sense of self. Regulations of extrinsic motivation can be placed on a continuum of a locus of causality, from internal to external. Extrinsic motivation with an internal locus of causality describes a more autonomous source of motivation, as opposed to that with an external locus which describes a more controlled source of motivation. The study of these forms of extrinsic motivation falls under Organismic Integration Theory (OIT), a sub-theory of SDT [33]. Amotivation refers to a complete lack of drive to act. SDT emphasises that stimulating intrinsic motivation is the most effective way to encourage contentment and effective learning in students [33].

Intrinsic motivation is fostered by creating environments which satisfy three basic psychological needs of students: autonomy, competence and relatedness [30,32–34]. Autonomy is the extent to which people are free to make their own choices. Competence is the perception of capability within a person. Relatedness encompasses the feeling of belonging, and being valued as part of a group. Motivation can also be impacted on by other circumstances that cannot be changed, such as age, gender, ethnicity, socioeconomic status, educational background, year of the curriculum and parent and teacher support [27].

Self-Determination Theory has been effectively applied to medical education to study student motivation and nurture student wellbeing [26–29]. Cultivating intrinsic motivation and autonomous forms for extrinsic motivation in students has been shown to lead to deeper learning and understanding of course material and promote reflection in learning and intention to engage in continuous professional development [27]. Productivity and higher cognitive processes were significantly boosted in students with greater autonomous forms motivation [35]. Higher levels of autonomous motivations, specifically intrinsic motivation, also has a positive relationship with academic grade in pre-clinical and clinical years [27,36,37]. Intrinsic motivation was also found to predict a greater advancement of final year medical students in their doctoral theses [38]. Hence educators should aim to foster these qualities of motivation in their students.

However, the majority of medical curriculum development has focused on introducing and modifying teaching practices to stimulate the cognitive and metacognitive aspects of learning, without purposefully addressing student motivation. Directly understanding the factors that foster autonomy, competence and relatedness will help educators develop intrinsic motivation in students. This understanding can also be applied to foster intrinsic motivation in medical students in other speciality rotations that face similar challenges to undergraduate ophthalmology education. Hence this study first aims to describe the variations in students’ perception of motivation in studying ophthalmology through their satisfactions of autonomy, competence and relatedness, and to highlight factors that impact on this. Next, this study aims to provide actionable recommendations to stimulate intrinsic motivation in ophthalmology education.

## Methods

### Study design

This study utilised a phenomenography methodology. Phenomenography examines the qualitatively different ways people experience, conceptualise, perceive and understand various aspects of a phenomenon of interest [39,40]. In this study, the phenomenon of motivation and the three basic psychological needs that influence motivation are described through medical students lived experiences using the theoretical lens of Self-Determination Theory.

In the description of their levels of motivation, students described experiences of the ophthalmology rotation to give insight into their type of motivation in relation to the satisfaction of the three basic psychological needs. In line with Marton [41], experience informs a person’s understanding of a phenomenon.

### Setting

Participants were medical students sampled from a single medical school which offers a 4-year graduate entry medical degree. Students undergo an ophthalmology rotation in their third year, which involves two face-to-face lectures, one half-day clinical skills tutorial, and three half-day clinical placements in hospital ophthalmology departments. These were interspersed between a longer 8-week General Practice rotation. Students were also required to watch online ophthalmology lectures and clinical skills videos, as well as complete optional worksheets. All students could reference their documented core curriculum which listed diseases, presentations and skills required to be learnt in their rotation.

Ophthalmology learning was assessed with a summative end of term exam, which consisted of multiple-choice questions in one section, and a second section of short-answer questions with image-based prompts. The end of year of written exam included two modified essay questions and one extended matching question in ophthalmology. There was also one ophthalmology station in students’ third year OSCE exam. Students are also asked to complete an ophthalmology procedural skill logbook during their clinical placements.

### Sampling

Purposive sampling was used in this study, and recruitment was voluntary. Students who had recently completed an ophthalmology placement were invited to participate in this study (*n* = 39). Sampling continued over a period of four months in 2021 and was ceased when diverse experiences regarding the phenomenon was obtained [41–44].

The sample size was determined by information power for data sufficiency [45]. The five dimensions of information power include the study aim, sample specificity, use of established theory, quality of dialogue, and analysis strategy. The adequacy of the sample size was continuously evaluated in the data collection and analysis process. Since our study aim was narrow, the sample was densely specific, SDT was used as a theoretical framework, quality of interview dialogue was strong, and a cross case analysis was performed, the attained information power of our sample was high, and data analysis could be ceased at 10 participants.

### Data collection

Participants engaged in a semi structured in-depth interview to determine aspects of their ophthalmology rotation that impacted on their motivation. This is the recommended structure in phenomenography studies as interviews are most suitable to the generation of student conceptions [45–48]. Interviews were carried out by DDCSD, who was not involved in any aspect of student assessment in their ophthalmology rotation. Interview questions were developed using the seven-step process outlined in the literature by Artino Jr and La Rochelle [49], which included conducting a literature review, initial focus groups interviews, developing interview questions, receiving feedback, cognitive interviews and a piloting stage. Interviews of students for this study were recorded and transcribed. All data, including students’ demographics, surveys, audio recordings and transcripts were de-identified. Questions used for guidance of semi structured interviews are listed in Supplementary List 1.

### Data analysis

Transcribed interviews were analysed according to the seven steps of analysis in phenomenography studies as outlined by Dahlgren and Fallsberg [50]. These steps and its procedure in this study are detailed in Table 1. Importantly, all steps described in Table 1 were carried out iteratively, using a constant comparative method. Analysis was carried out with the NVivo software (QRS NUB*IST Vivo).

**Table 1.** Steps of analysis in phenomenography research as outlined by Dahlgren and Fallsberg [50] with elaboration of the procedure in this study. DDCSD, HR and SC are authors of the present study.

Steps	Description	Procedure in this study
1. Familiarisation	Reading through all interviews	All interviews were transcribed and read by DDCSD
2. Compilation	Organising student responses according to each interview question and identifying significant elements in responses	Transcripts organised and significant elements of each answer noted by DDCSD
3. Condensation	Reducing answers to discern central meaning units	Preliminary meaning units of answers of two transcripts identified by DDCSD Discussion and agreement between DDCSD, HR and SC on meaning units of initial two transcripts. Disagreement was resolved by discussion between authors and revisiting the transcript data. DDCSD completed condensation of remainder transcripts. Meaning units of all transcripts were confirmed independently by DDCS, HR and SC. Disagreement was resolved by discussion between authors and revisiting the transcript data. Meaning units often identified factors that affected motivation
4. Preliminary grouping	Allocating similar meaning unit into groups of meaning	Arranging meaning units of answers into preliminary dimensions of variation by DDSD, maintaining factors that affected motivation previously identified Hierarchical conceptions within these dimensions of variation emerged, and were noted by DDCSD Preliminary dimensions of variation and hierarchical conceptions were confirmed independently by HR and SC
5. Preliminary comparison	Differentiating groups of meaning, ensuring each is unique	DDCSD carefully delineated each dimension of variation and conceptions to ensure each was specific and unique Meaning units were independently scrutinised by DDCSD, HR and SC to ensure they belonged to specific dimension of variation and conceptions Refinement of preliminary grouping was Factors that affected motivation were differentiated and their relationship to each dimension and conception was identified The above was done iteratively by DDCSD, HR and SC independently until a more optimal distinction of meaning units, dimensions and conceptions could not be realised.
6. Naming	Assigning meaningful labels to each group	Dimension of variation and conceptions were named according to the terminology of Self-Determination Theory by DDCSD Factors that affected motivation were named to represent their true meaning by DDCSD All names were reviewed and agreed upon independently by DDCSD, HR and SC Steps 1–6 were done iteratively, until researchers deemed there to be true distinctions between names of factors of motivation, dimensions and conceptions and their associated meanings.
7. Contrastive comparison	Contrasting and describing groups of meaning according to their essence of meaning and identifying relationships between them.	Emergence of an outcome space through study of the relationships between conceptions in dimension of variation, noted by DDCSD Factors that affected motivation were represented according to their impact on each their relationship to each dimension and conception was identified, noted by DDCSD The outcomes space and factors that affected motivation carefully scrutinised independently by DDCSD, HR and SC

### Outcome space

Analysis of the transcripts lead to the formation of an outcomes space. An outcomes space is defined as a logically structured diagrammatic representation of phenomenography results [48,51]. An outcome space intends to describe each aspect of the students’ perception of a phenomenon, with detailed quotes to support each aspect [52,53]. This leads to deeper understanding of students perceptions and the similarities and differences between them [54]. A phenomenon is represented in distinct dimensions of variations. Dimensions of variation are defined as distinct qualities of a phenomenon that are realised by students [55]. These dimensions describe the ways in which students experience different aspects of motivation. The full range of dimensions of variation is encompassed in a set of conceptions. Conceptions represent students’ expanding understanding of underlying dimensions [55]. These are hierarchically related and inclusive [56]. In this study, these dimensions of perceptions of motivation were arranged hierarchically into conceptions, according to the terminology of SDT, and formed the basis of the outcome space [57].

Variation in experiences reflect students’ internal and external horizons of awareness [57]. The internal horizon discerns the aspects of a phenomenon, in this case motivation, and how they relate to each other to coalesce into an experience. The external horizon discerns an experience from its context, representing aspects of the phenomena yet to be realised. A less sophisticated experience of motivation would entail a narrow internal horizon of experience (lesser quality motivation) and a wider external horizon of experience (greater aspects of motivation yet to be realised) [58]. In this study, the arrangement of dimension of variations and conceptions represent different levels of awareness and non-awareness of critical aspects of a phenomenon [59].

### Trustworthiness

The trustworthiness of this study was ensured by first clearly describing the context of the study [60]. Academic rigour in the methodology was maintained by abiding to phenomenography principles described and validated in the literature [50,61,62]. Reflexivity was addressed by ensuring that aims of the study was informed by thorough literature review and that researchers engaged in continuous discussion and revisitation of data to ensure student voice informed interpretations [55]. In this study, credibility was maintained by using detailed quotes to justify all findings [63].

## Results

Respondents were third year students from a single tertiary educational institution who had most recently completed an ophthalmology rotation as part of a four-year post-graduate program. Of 39 total eligible students, ten students were interviewed (mean age of 23 years, with six students identifying as male, and four identifying as female).

## Outcome space: motivation space in ophthalmology education (MSOE)

Using a constant comparative approach to analyse student interviews, 3 main dimensions were identified that could be used to describe the aspects of perception of student motivation: autonomy, competence and relatedness. Importantly, these dimensions do not represent levels of 3 basic psychological needs in students, but how students perceive them. These dimensions of variations could be arranged hierarchically into conceptions of motivation outlined on the continuum of quality of motivation in SDT, and formed the basis of the outcome space in Table 2. Hence the outcome space represents the variation of realisation and perceptions of different levels of the 3 basic psychological needs by students. The outcome space was termed Motivation Space in Ophthalmology Education (MSOE).

**Table 2.** Motivation space in ophthalmology education (MSOE): dimensions of variation and hierarchical conceptions of students’ experiences.

Dimension of variation	Amotivation	External locus extrinsic motivation	Internal locus extrinsic motivation	Intrinsic motivation
**Autonomy**: *desire to initiate one’s own behaviour*	**A1**: No perception of control during their ophthalmology rotation	**A2**: Desire to act according to a structured timetable and ophthalmology curriculum	**A3**: Feeling of uninhibited exploration of the ophthalmology curriculum	**A4**: Innate interest to engage in activities within and beyond the ophthalmology curriculum
**Competence**: *desire to feel effective*	**C1**: Feeling of inability to achieve proficiency of ophthalmology	**C2**: Desire to learn for fulfilling set curriculum standards	**C3**: Drive to understand core concepts in ophthalmology	**C4**: Constant desire to achieve, maintain and enhance confidence and effectiveness in their ophthalmology knowledge and skill
**Relatedness**: *desire to belong and feel connected*	**R1**: Perception of alienation from mentors and ophthalmology as a discipline	**R2**: Accepting of mentors as authoritative and paternalistic	**R3**: Perception of belonging within the ophthalmology rotation as in the role of a student	**R4**: Strong sense of acceptance and connection to peers and mentors during their ophthalmology rotation

The varying perceptions of autonomy, competence and relatedness seemed to foster different qualities of motivation in students. This informed the conceptions in the MSOE. Students who did not perceive their psychological needs to be satisfied often expressed amotivation. Students who found that their basic psychological needs were beginning to be addressed by the ophthalmology curriculum expressed extrinsic motivation with an external locus of causality, which describes the second conception. The third conception describes students who were extrinsically motivated with an internalised locus of causality. These students perceived the ophthalmology rotation to meet their basic psychological needs. Students who were intrinsically motivated displayed high levels of autonomy, competence and relatedness, which was fostered by and applied to all aspects of their ophthalmology rotation. This was captured in the fourth conception.

Variation in experiences in individual students are reflected in their internal and external horizons of awareness [57]. The internal horizon in the MSOE refers to how students experience the 3 basic psychological needs, and how these work in relation to each other to impact on the quality of motivation. The external horizon of awareness of students alludes to the context of motivation, and the wider extent and quality of motivation that is yet to be realised.

## Autonomy

Autonomy refers to the desire for people to feel that they are their own source of behaviour or action [30]. Students appreciated varying perceptions of autonomy during their ophthalmology placement. Variations in perceptions of autonomy originated from the perceived limits of students’ freedoms. To one end, some students described a lack of autonomy during their ophthalmology rotation, which fostered a sense of amotivation in students.

‘I felt like I was mostly just sitting in the corner and occasionally they would invite me to go and look at things. Otherwise, I just basically watched them and then got out of there. It was not great. You can imagine I had little motivation to study after that experience’. (Student 6)

This perception contributed to the conception of autonomy in the first column of the MSOE. Conceptions of autonomy increased in complexity as students perceived an expanding sense of freedom. Some students described their perception of autonomy to be limited to the confines of curriculum rules. This fostered extrinsic motivation, with curriculum requirements serving as students’ external locus of causality.

‘There were very specific requirements to get a lot of skills signed off in a particular way, and to examine a certain number of patients. Sometimes that was difficult because there wasn’t any flexibility in cases where you might not see that many of a particular type of patient, but I had to get it done so most of my energy was devoted to completing those requirements’. (Student 10)

However, seven of the ten students who participated in this study perceived high levels of autonomy within their ophthalmology rotation. This was generally attributed to the support of staff and mentors, as well as the availability of online lectures.

‘So, my autonomy was definitely supported by the fact that all the lectures were easily available online. That was really, really useful, especially because some of my placements were quite far away, I would have that playing whilst I’m on the commute. That was great. I could be watching lectures at my own pace, and I felt like I was learning in a self-directed way’. (Student 3)

This level of autonomy fostered an internalised regulation of motivation, as students accepted and valued behavioural goals such as completing lecture material. Even though students displayed an internal locus of causality, motivation was still extrinsic as behaviour was driven by outcomes external to the behaviour itself.

Finally, some students found their autonomy to be completely satisfied, where their study was motivated by the freedom to explore the discipline of ophthalmology.

‘The opportunity to have my own room and see patients myself, present them to the registrar and get a lot of bedside teaching was invaluable. That freedom was very motivating for me in my studies. I even observed quite a few surgeries even though it wasn’t specifically in the curriculum’. (Student 7)

Additionally, these intrinsically motivated students often chose to engage in optional activities out of their own stated interest. This exemplifies how intrinsically motivation drives action for the sake of its own enjoyment.

## Competence

Competence refers to the desire for one to perceive themselves as effective in tasks that they perform [30]. It is important to note that competence in SDT is not a measure of skill or ability, but rather student’s perception of confidence and their effectiveness. Variations in perceptions of competence arose from students’ perceived confidence, and their desire to attain a deeper understanding of the discipline of ophthalmology and the ophthalmology curriculum. Students who lacked perceptions of competence in their rotation experienced amotivation to explore the topics within the ophthalmology curriculum.

‘When I find things difficult, I suppose that I can really assure myself that’s a bit more niche, so if I don’t know how to do something … we don’t need to know most of it anyway’. (Student 6)

For some students, the grade standards of the curriculum acted as the external locus that fostered their extrinsic motivation, in line with the second conception of this study. Students perceived their competence to meet the minimum grade required to progress through the rotation, and often did not express a desire to exceed this standard.

‘Mainly I was studying to just try to make sure that I passed the rotation and get good marks’. (Student 10)

A higher conception of competence was expressed by some students, who had a drive to attain a deeper understanding of core conditions and presentations listed in the ophthalmology curriculum. This represented an internal locus of causality. These students were often encouraged by an organised curriculum and constructive feedback. However, these students were mainly extrinsically motivated to achieve academic proficiency.

‘Ophthalmology is part of our assessment. We have an OSCE station on it and it is in our examinations, so that was a strong factor for my motivation too’. (Student 1)

Intrinsically motivated students perceived high levels of competence or a drive to attain competence, however this was not only limited to their ophthalmology rotation.

‘I’m always very keen to learn about new systems. I feel that it helps me to grow into a better doctor’. (Student 1)

## Relatedness

Relatedness is defined as the desire for individuals to have an interpersonal connection and a sense of belongingness to other significant individuals and a significant community [30]. Some students did not perceive a sense of relatedness with their mentors in their ophthalmology rotation.

‘On placement, my consultant was very detached. They didn’t seem too interested in students in general. That was quite off-putting’. (Student 2)

This contributed to a sense of alienation, common among students experiencing amotivation in the first conception. Extrinsically motivated students experienced a greater sense of relatedness compared to those who lacked motivation. Motivation that arose from an external locus, as described in the second conception, was generally fostered by a sense of relatedness towards mentors who provided guidance and structure. Students often described this guidance to be provided in a paternalistic fashion, which maintained a sense of hierarchy between student and teacher.

‘The registrar was really good at giving instructions. It made it easy for me to just follow along, step -by-step’. (Student 6)

This sense of hierarchy was maintained in those students who were extrinsically motivated with an internal locus of causality. However, the process of internalisation was stimulated by a sense of welcome and inclusion experienced by students, which differentiates the second and third conception.

‘Helping assist nurses in the measurement of power, putting in dilating eye drops, for example, and with the registrars making us feel really included, especially as they were allowing us to look at a slit lamps and report back to them what we see, in that regard, we were treated as part of the team. And it’s always nice to be part of team, that helps in motivation and to learn’. (Student 2)

Here, students felt that they played a valued part in patient care, fostering a sense of relatedness. Intrinsically motivated students had a complete sense of relatedness with peers and mentors in their ophthalmology rotation. A sense of respect and equality pervaded their learning experience, often dissolving the confines of hierarchy.

‘The culture at the hospital I was at was really good. I think every single person made me feel part of the team: the receptionists, the nurses, the doctors. It feels like you are almost working alongside peers and that we are all learning’. (Student 7)

These students maintained the perception of being a valued team member and contributing to patient care, identifying and relating closely with the roles of their clinical and allied health mentors.

## Factors affecting motivation

**Figure 2.**
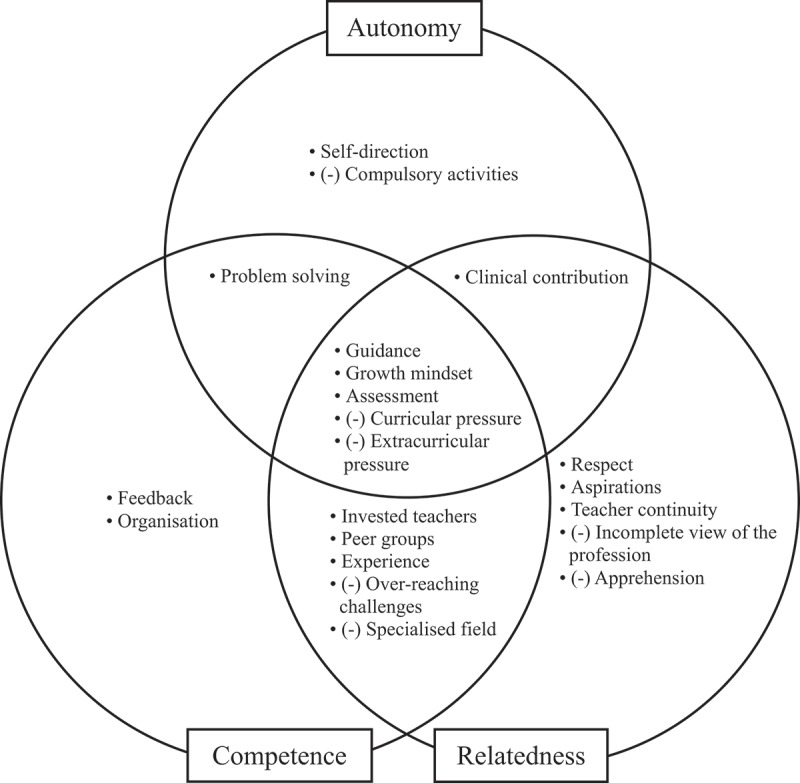
Factors that impact on students’ perception of autonomy, competence and relatedness, (-) indicates factors with a negative impact.

A secondary aim of this study was to describe factors that were highlighted by students to have impacted on their motivation by affecting their perception of their autonomy, competence and relatedness. Through student interviews, 21 factors were identified and are displayed in Figure 2. Certain factors contributed more heavily to a certain conception of one or more particular psychological need, displayed in Supplementary Table S1 along with definitions of each factor and supporting quotes. Nine factors (43%) affected only one of the three basic psychological needs, seven (33%) affected two needs, and five (24%) affected all three needs. Of the 21 factors, 17 (81%) were influenced by students’ learning environment.

Some factors were specific to fostering either autonomy, competence or relatedness in a predictable manner, including self-direction, feedback, organisation, respect, aspirations and teacher continuity. Other factors that detracted from one of either autonomy or relatedness included compulsory activities, incomplete views of the profession and apprehension.

Some factors contributed to fostering or limiting more than one of the three basic psychological needs, displayed in the intersections of the Venn diagram. Importantly, students identified five over-arching factors that impacted perceptions of all three basic psychological needs. These include guidance, growth mindset, assessment, curricular pressure and extracurricular pressure.

Guidance refers to the concept of providing non-paternalistic and non-judgmental support to students. This support is often directed by students’ individual needs. Guidance is also often provided in a safe setting where students feel comfortable, including away from patients to not be perceived as incompetent.

Students who exhibited a growth mindset also described high levels of perceived autonomy, competence and relatedness. A growth mindset is defined as a belief that one’s abilities can be fostered through their own effort and learning [64]. Students often exhibit this frame of mind in most aspects of their curricular and extracurricular activities.

The types and quantity of prescribed assessment played a complex role in impacting on students’ perception of the three basic psychological needs. Some students perceived their autonomy to be affected by the knowledge that assessments draw primarily from lecture content.

‘I wouldn’t say ophthalmology was the most autonomous rotation just because we knew that our assessments were coming directly from those lecture slides. So again, I didn’t feel motivated to do further study myself during clinic’. (Student 6)

Even though these students did appreciate a moderate level of autonomy, they felt restricted by the confines of their recommended lecture list, contributing to the second conception of autonomy. Additionally, a minority of students expressed that there were too many procedural skills requirements to be signed off in the rotation, which limited their autonomy.

The impact of assessment on competence depended on students’ perception of the difficulty of assessment. Students who perceived assessments as appropriately difficult often perceived a high level of competence that contributed to their extrinsic motivation. However, students who perceived assessments as beyond their capabilities often displayed reduced perceived competence and were discouraged from further study.

Assessment modality was also an important factor for perceived competence and student motivation. For example, the end of term visually aided exam was perceived to negatively impact on their exam performance due to the set time limit of one minute per question and deterred some from studying for the ophthalmology component of their final exam. Assessment requirements also contributed to student’s sense alienation from mentors, which reduced their perception of relatedness and hence contributed to amotivation.

Curriculum pressures experienced by students is a product of curriculum requirements and time constraints. The short duration of the rotation contributed to student’s perception of pressure which impacted on the autonomy that students perceived they had to explore areas of interest beyond the essential curriculum requirements.

‘I had only two clinics so that made it of course a bit more difficult to try to branch out and do my own things’. (Student 3)

Additionally, students felt that increased pressure resulted in a reduced exposure to patients. This meant less experience in managing ophthalmic disease and reduced perceptions of competence.

‘I think a whole week of ophthalmology, instead of a few half days would be beneficial. That’ll give us more exposure especially to theatres, clinics, and more presentations of disease’. (Student 1)

Relatedness was negatively impacted by perceptions of pressure and time constraints within the curriculum by limiting tutor interactions.

‘But it is a bit busy. So, it can sometimes feel like you’re getting in the way, you don’t get as much learning out of it’. (Student 2)

Hence, the negative impact of pressure on perceptions of autonomy, competence and relatedness contributed to students’ amotivation, which is the first conception of motivation in the MSOE.

Extracurricular pressures contributed to time pressure, which reduced their motivation to engage with the ophthalmology course. This was true for extracurricular academic activities part of the wider medical school course requirements. Lifestyle factors external to medical school also played a large role in modulating student motivation to learn.

In summary, there were a multitude of factors that students identified that foster or hinder their basic psychological needs, mostly arising from their learning environment. Of these factors, guidance, growth mindset, assessment, curricular and extracurricular pressure affected all three basic psychological needs of students.

## Discussion

### Variations in motivation and basic psychological needs

Motivation in SDT is described as being on a continuum from amotivation to forms of extrinsic motivation and finally to intrinsic motivation [34,65]. This continuum of amotivation to intrinsic motivation is also found in the variation of motivation presented within the MSOE (Table 2). Additionally, the findings of this study emphasises that motivation varies in quality and not just in quantity, concurring with SDT in that it is not the amount of motivation rather the type of motivation that has the greatest impact on student satisfaction [34,65].

This study is the first to provide a rich description of the basic psychological needs within each stage of motivation in the context of ophthalmology education. The connections between variations in the perceptions of the three basic psychological needs and quality of motivation in students as presented in the MSOE (Table 2) has been alluded to in previous studies. Several studies on SDT report that intrinsic motivation and autonomous forms of extrinsic motivation is fostered by supporting autonomy, competence and relatedness [31,66]. For example, Kusurkar et al. [27] describes motivation as a dependent variable on autonomy, competence and relatedness, and as an independent variable on learning outcomes and student satisfaction. This aligns with students’ perception of motivation as a subject of their level of satisfaction of the basic psychological needs in our study. Additionally, in a review of the SDT literature, ten Cate et al. [30] outlined that the fulfilment of the basic psychological needs plays a key role in allowing students to progress from an external to an internal locus of extrinsic motivation.

### Factors impacting autonomy, competence and relatedness

The factors identified in Figure 2 and Supplementary Table S1 have varying effects on autonomy, competence and relatedness. Most factors did not impact on all basic psychological needs together, and hence can be considered as low-level impacting factors. Some of these low-level factors have been alluded to in previous studies. For example, Hartnett [67] conducted a study on the impact of online teaching methods on learner motivation through the lens of SDT, describing factors that undermine autonomy (high workload, assessment pressure and perceptions of lacking relevance), competence (unclear guidelines, insufficient guidance and feedback) and relatedness (poor communication). Many similarities can be drawn between these factors and the low-level factors outlined in our study. In a related study, van der Burgt et al. [68] highlighted time pressure and organization, amongst other factors, to impact motivation of medical specialists for practice. The low-level factors outlined in the present study and previous studies can be appropriately addressed by educators. Hence, they have the potential to foster individual psychological needs to encourage intrinsic motivation.

The majority of these impacting factors identified in this study are contextual, which concurs with previous studies that highlights the impact of the learning environment on perceptions of autonomy, competence and relatedness [27,69,70]. In addition, some factors seem to relate to certain conceptions of either autonomy, competence or relatedness as seen in Supplementary Table S1 (for example, respect cultivates the highest conception of relatedness, whilst teacher continuity cultivates the second conception of relatedness). This indicates that some factors have the potential to stimulate a greater quality of motivation than others, which should be considered when educators address these factors in their ophthalmology teaching.

Importantly, this study adds to the SDT literature in identifying five over-arching factors that impacts all perceptions of the three basic psychological needs. These factors are guidance, growth mindset, assessment, curricular pressure and extracurricular pressure. Some of these factors are internal to students, such as a growth mindset. This is defined as a belief that one’s abilities and talents can be developed through sustained and improved effort, learning, and perseverance, compared to a fixed or entity mindset where it cannot [64]. This mindset is reported as being central to SDT, where a core assumption is that humans have natural growth tendencies to attain a sense of intrinsic motivation, which are supported by the fulfilment of basic psychological needs [30,33,71]. Some over-arching factors are external to the student such as guidance, pressure and assessment. These are common themes incorporated into autonomy supportive teaching, which is defined as a positive interpersonal relationship between students and teachers, where teaching is delivered with non-judgmental support and minimal control [72]. This style of teaching is central to the application of SDT in educational settings, and has been shown to foster all three basic psychological needs and student wellbeing [27,29,30,72]. For example, a study by Neufeld and Malin [69] showed that student perceptions of autonomy-support significantly predicted better student perceived wellbeing, heavily mediated by the satisfaction of the three basic psychological needs.

### Application in ophthalmology education

The MSOE (Table 2) may be adapted to gauge student motivation and may be developed as a rubric to evaluate the psychological need satisfaction within an ophthalmology rotation. This will allow educators to conceptualise the effectiveness of the affective component of learning within their programs.

The factors mentioned in Figure 2 and Supplementary Table S1 provide actionable recommendations to foster intrinsic motivation in students within an ophthalmology rotation (**Box 1**). Most importantly, ophthalmology educators should prioritise providing guidance and minimising pressure in their teaching practices. The seven recommendations within Box 1 address curriculum design, clinical placements, teaching styles and teacher wellbeing and may serve as a basis for ophthalmology educators to identify and address factors within their curriculum that impact on intrinsic motivation of learners. These recommendations may also positively impact aspects of learning other than the affective. For example, integrating self-direction and problem solving in medical education has been shown to enhance the cognitive aspects of learning [17,72–75]. Additionally, assigning appropriate assessments allows for the cognitive process of test-enhanced learning [76]. Providing constructive feedback also improves educational outcomes by metacognitive learning [77].

**Table:** 

**Box 1**: Recommendations for educators to foster intrinsic motivation in medical students studying undergraduate ophthalmology Organise a curriculum with ample of opportunity for supported self-direction and problem solving Listen and act to minimise perceptions of pressure and apprehension in students Allow students to actively contribute to patient care in clinical placements Assign appropriate assessments that purposefully reflect curriculum goals Provide constructive formal and informal feedback on students’ ophthalmic knowledge and procedural skills in-class and placements Provide non-judgemental guidance in lectures, tutorials, workshops, clinical placements and informally Cultivate motivation and well-being within yourself to ensure you are best positioned to invest in and respect your students

### Limitations

Since all students were voluntarily recruited from a single academic institution, they have similar experiences of ophthalmology education, and may share interests in research and education [78]. To address this, sampling continued until diverse experiences of motivation were obtained. Additionally, interview questions were focused on students’ psychological response to learning experiences, which allowed for the development of a more insightful outcome space.

Student responses, dependent on their individual experience and context, were used to interpret the abstract phenomena of motivation that may not reflect individual responses [79,80]. This was an inherent potential limitation of the qualitative study design. However, this was addressed by having a semi-structured interview style where subjects were encouraged to explore their conceptions of motivation. Interviewees were also encouraged to explore the reasons behind such conceptions. This served to minimise extrapolation in the data analysis process.

In the process of data analysis, the experiences and context of the primary researcher will have had an influence on their interpretation of the conceptions presented by students. This was addressed by careful scrutinisation of these interpretations and analyses by other members of the research team to ensure the trustworthiness of the analysis. However, due to the relative homogeneity of the research team, there remains potential for alternative interpretation of the interview data.

## Conclusions

The findings of this study illustrate the variation of realisation and perceptions of different levels of the three basic psychological needs of autonomy, competence and relatedness by participants that influence their motivation for learning ophthalmology. Additionally, this study describes tangible factors that impact on their psychological needs. This insight should be considered by ophthalmology educators when adapting teaching practices and curriculum designs to foster intrinsic motivation in their students. If applied into education practice, the recommendations made in this study may improve the efficiency and effectiveness of not only undergraduate ophthalmology education, but other speciality disciplines that face similar challenges. This study encourages educators to adopt a holistic approach to medical education, where the affective aspects of learning are addressed equally with the cognitive and metacognitive.

## Supplementary Material

Supplemental Material

## Data Availability

The datasets generated during the current study are available from the corresponding author on reasonable request.

## Authors’ information

Deepaysh D.C.S. Dutt, BSc, MD is amedical doctor completing his Master of Health Professions Education at The University of Western Australia.

Hessom Razavi, MBBS, BSc, MSc (Hons),FRCOphth, FRANZCO is a consultant ophthalmologist at the Lions Eye Institute and the coordinator for ophthalmology teaching at The University of WesternAustralia.

Sandra E. Carr, BSc, MPH, PhD, SFHEA,is Head, Discipline of Health Professions Education at The University ofWestern Australia.
