# Supplementary material for: Self-determination theory in ophthalmology education: factors influencing autonomy, competence and relatedness in medical students
Source: Med Educ Online. 2023 Sep 20;28(1):2258633. doi: 10.1080/10872981.2023.2258633 (PMC10512850; doi:10.1080/10872981.2023.2258633)
Supplement: Supplemental Material [file ZMEO_A_2258633_SM7246.docx]

**Supplementary Material**

**Supplementary List 1:** Interview questions

1. Are you a medical student? What year are you in?
2. Tell me about your motivation during your ophthalmology placement in general.
3. How was your autonomy hindered or supported in the ophthalmology rotation (including lectures, tutorials and placements), and how did this impact on your motivation levels to study ophthalmology?
4. What aspects of the rotation made you feel part of a team or excluded from a team, and how did this have an impact on your motivation?
5. What aspects of the rotations made you feel more/less competent with your work, and how did this affect your motivation?
6. What did you enjoy most about your ophthalmology rotation?
7. What do you think could be improved?

**Supplementary Table 1:** Meaning and examples of factors that impact on students’ perception of autonomy, competence and relatedness

| **Theme** | | | **Factor** | **Meaning** | **Quotes** |
| --- | --- | --- | --- | --- | --- |
| **A** | **C** | **R** |  |  |  |
| **A3** |  |  | Self-direction | Allowing for students to engage in course material freely and accessibility | “My autonomy was definitely supported by the fact that all the lectures were easily available online. That was really, really useful, especially because some of my placements were quite far away, I would have that playing whilst I'm on the commute. That was great. I could be watching lectures at my own pace, and I felt like I was learning in a self-directed way” (Student 3) |
| **A4** | **C4** |  | Problem solving | Opportunity to consolidate information to solve clinical problems | “So, bringing the learning through the tutorials and the lectures into our history taking and clinical management knowledge, then doing all the exams by ourselves, using the complicated slit lamps and the ophthalmoscope, and having a good look into the eyes and putting them all together to come up with possible differential diagnoses and figuring out how to go on from there at my own pace with the content online. That’s something that I enjoyed the most in the course.” (Student 4) |
| **A1** |  |  | Compulsory activities | Lectures, tutorials and placements where attendance was compulsory | “In-person lectures were compulsory. I thought the whole thing about the post-grad degree is that we're supposed to be independent learners and to go to things if they suit us and not go to things if like they don't suit us. Not everyone learns well from lectures.” (Student 6) |
|  | **C3** |  | Feedback | Constructive criticism both formally (through assessments) or informally (through mentors and peers) | “In clinic I got some good feedback as well. One of assessors said that my anatomy knowledge was very strong for a student so that made me feel good and made me feel like I wanted to study more.” (Student 2) |
|  | **C3** |  | Curriculum Organisation | Well planned learning activities and opportunities coherently integrated into an ophthalmology rotation within a medical curriculum. Activities are generally related and progressive not repetitive. | “We had some level of basic anatomy and physiology knowledge from years 1 and 2 and to then have the autonomy in year 3 to have ample practise to build on and actually use that knowledge was really motivating.” (Student 6) |
|  | **C1** | **R1** | Over-reaching challenges | Providing learning experiences or challenges that are far beyond students perceived level of expertise | “When tutors throw you in difficult clinical scenarios it certainly negatively impacts on motivation… if they don't take time to necessarily help you with the inferential steps that they simply take for granted it really makes one kind of come to erroneous conclusions.” (Student 9) |
| **A3** |  | **R3** | Clinical contribution | Contributing to patient care | “Helping assist nurses in the measurement of power, putting in dilating eye drops, for example, and with the registrars making us feel really included, especially as they were allowing us to look at a slit lamps and report back to them what we see, in that regard, we were treated as part of the team. And it's always nice to be part of team, that helps in motivation and to learn.” (Student 2) |
|  |  | **R4** | Respect | Students’ perception of being respected by mentors | “I am the sort of person that asks a lot of questions, probably annoyingly so, and they (clinicians) would answer some but sometimes they would shut me down by saying that's way above your level, don't bother. So, you sort of reach a point where you're like, okay, maybe I just shouldn't ask. I think that aspect probably made me feel like I wasn't really supported and obviously wasn't very good for your study motivation.” (Student 3) |
|  | **C3** | **R3** | Peer groups | Allowing for student collaboration | “I think that what made me feel most competent was revising the lectures and then going through content with like peers” (Student 5) |
|  | **C3** | **R4** | Invested teachers | Teachers motivated to foster student learning | “The advanced trainee in ophthalmology at the clinic was a very, very great teacher who was actually quite invested in teaching and sharing knowledge, which was great. My motivation and competence were really a function of the advanced trainee or registrar taking the time to sit with me and actually work through the cases with me and allow me to use the slit lamp as well. In short, basically everything about the learning is just a lot more fun and easier when you have teachers who are interested in your learning.” (Student 9) |
|  | **C3** | **R4** | Experience | Prior exposure to ophthalmology | “So, it's pretty exciting to learn about it and because I've worked as an optical dispenser. So, like I've seen lots of things and had bits and pieces of teaching before.” (Student 3) |
|  |  | **R4** | Future aspirations | Career ambitions or interests that encourage ophthalmology expertise | “I find ophthalmology interesting, and because I want to do ED or GP or something in that area in the future, I thought, you know, it's important that I have a good understanding of basic ophthalmology.” (Student 3) |
|  |  | **R2** | Teacher continuity | Engaging with one mentor over time | “Placements are three half days, and you sit in with a different doctor every session, so, we didn't really have the opportunity to really connect with our team.” (Student 7) |
|  |  | **R1** | Incomplete view of the profession | Students not understanding the role of ophthalmology within the medical profession | “We only did outpatient clinic. In retrospect, I think it'll be valuable to see inpatient like ward consults and also a chance to go to theatre. I think that would give me a more holistic understanding of what being part of an ophthalmology team is because for me, outpatient clinic was really valuable study, but I still don't really understand what the day to day of an ophthalmologist is. So that limited my interest in the field.” (Student 1) |
|  |  | **R1** | Apprehension | Trepidation due to adopting certain pre-conceptions | “Before my rotation, everyone was like, there's no real points studying this properly, because the assessments are so hard at the end, you're probably not going to do well, even if you study.” (Student 6) |
|  | **C1** | **R1** | Specialised field | Perceptions of ophthalmology as a niche speciality lacking interdisciplinary relevance | “Anything where you're just put in a room, let's say the nurses, like the pre-clinic kind of work up when you're put in a room with a lot of big machines and you watch people get their photos of their retinas taken, and do random tests that mean nothing to you, that makes you feel incompetent. Certainly, if that's only point of contact or experience you had with ophthalmology, you might think that it's far beyond you.” (Student 8) |
| **A4** | **C4** | **R4** | Guidance | Providing non-judgmental support and tutorage according to the student requirements | “The registrars I was with, with sort of every patient they would have me to look through the slit lamp and then explain some stuff that was on the slit lamp when the patient left, that was really helpful. That sort of support and guidance in a safe environment was really reassuring and motivating” (Student 9) |
| **A4** | **C4** | **R4** | Growth mindset | A purposeful drive for self- development | “I'm always very keen to learn about new systems. I feel that helps me to grow into a better doctor.” (Student 1) |
| **A2** | **C2** | **R1** | Assessment | Students’ perception of the difficulty, number, and type of assessment. | “There were very specific requirements to get a lot of skills signed off in a particular way, and to examine a certain number of patients. Sometimes that was difficult because there wasn't any flexibility in cases where you might not see that many of a particular type of patient, but I had to get it done so most of my energy was devoted to completing those requirements.” (Student 10) |
| **A1** | **C1** | **R1** | Curricular Pressure | Factors related to the ophthalmology curriculum that contributed to stress | “Having a bit more time on the rotation would just make things a little bit less stressful on students, so they don’t feel like *“Oh, I have to learn all of ophthalmology and get everything signed off”* and they'll be able to kind of ease into learning a bit more.” (Student 5) |
| **A1** | **C1** | **R1** | Extracurricular pressure | Factors external to medical school that contributed to stress | “It was a kind of hectic time of year and life happened. I was part of organising quite a big event around that time and I had an injury and all sorts of things. Priorities changed, which was unfortunate… I think it's the whole external environment like the other things I had going on was probably not the best in terms of what it's supporting my motivation to study” (Student 4) |
